# Spontaneous Deceleration and Acceleration of Growth Rate in Medullary Thyroid Carcinomas Suggested by Changes in Calcitonin Doubling Times Over Long-Term Surveillance

**DOI:** 10.1007/s00268-018-4789-1

**Published:** 2018-09-18

**Authors:** Akira Miyauchi, Takumi Kudo, Minoru Kihara, Hitomi Oda, Yasuhiro Ito, Akihiro Miya

**Affiliations:** 1 Department of Surgery, Kuma Hospital, 8-2-35 Shimoyamate-dori, Chuo-ku, Kobe, 650-0011 Japan; 2 Department of Internal Medicine, Kuma Hospital, Kobe, Japan

## Abstract

**Background:**

Based on our long-term observation of medullary thyroid carcinoma (MTC) patients, we hypothesized that some MTCs have spontaneous deceleration or regression of tumor growth over a long term and that a minority may acquire growth acceleration. We thus compared the calcitonin doubling time (Ct-DT) in the earlier and later half-periods of MTC patients’ postoperative course.

**Methods:**

We followed 26 MTC patients (14 hereditary and 12 sporadic MTCs) with postoperative hypercalcitoninemia with periodic measurements of serum calcitonin (Ct) for >10 years without major interventions. The median period of Ct measurements was 18.3 years (range 10.6–30.2 years). We divided the individual patients’ study periods into the earlier and later halves and calculated the Ct-DTs for both periods.

**Results:**

In the hereditary group, the Ct-DT in the later half-period (Later-Ct-DT) was significantly longer than that in the earlier half-period (Earlier-Ct-DT) (median 20.0 years vs. 7.1 years, *p* = 0.013). These values in the sporadic group were 20.0 years versus 11.1 years, respectively (*p *=0.774). Twelve patients (seven hereditary and five sporadic) had Later-Ct-DTs significantly longer than their Earlier-Ct-DTs (median 27.4 years vs. 4.9 years) and good prognoses. Two patients (one hereditary, one sporadic) had Later-Ct-DTs significantly shorter than their Earlier-Ct-DTs, and both developed structural recurrence and died of the disease.

**Conclusion:**

Many of the hereditary and some of the sporadic MTC patients had elongated Ct-DTs over a long period, suggesting spontaneous deceleration and regression of tumor growth. A minority of the MTC patients showed Ct-DT shortening, suggesting tumor growth acceleration.

## Introduction

Medullary thyroid carcinoma (MTC) is a rather rare malignancy arising from calcitonin-producing C-cells of the thyroid [1, 2]. MTCs may arise as a component of multiple endocrine neoplasia type 2A (MEN 2A), type 2B (MEN 2B), or familial MTC (FMTC). Patients with these hereditary syndromes carry activating germ line mutations of the *RET* proto-oncogene [3, 4]. MTCs may also occur as sporadic non-hereditary tumors. MTCs secrete calcitonin, and the vast majority of MTCs also produce carcinoembryonic antigen (CEA) [5, 6], the reason for which is not yet known. Calcitonin and CEA serve as very good serum tumor markers of MTCs.

MTCs tend to spread to the regional lymph nodes and distant organs. Compared to sporadic MTCs, hereditary MTCs tend to arise at younger ages [2, 7]. Nodal metastases and postoperative hypercalcitoninemia indicating biochemically persistent disease are common even in young patients with hereditary MTCs. The MTCs in MEN 2B have an aggressive nature, and their prognosis is generally poor [8]. The prognosis of hereditary MTC in MEN 2A and FMTC, however, is generally good [2, 7].

We observed and reported in 1984 that the changes in serum calcitonin levels over time in patients with MTCs who had persistent hypercalcitoninemia postoperatively were exponential and that the calcitonin doubling time (Ct-DT) was a strong prognostic indicator [9]. Other researchers confirmed that the Ct-DT and the CEA-DT are very strong prognostic indicators of MTCs [10, 11]. Collins et al. [12] studied the changes in the tumor volumes of pulmonary metastases over time and proposed the concept that the growth of human tumors is exponential. The changes in serum calcitonin and CEA fit quite well with this concept [9, 13].

It is well known that over a long-term surveillance, the biological nature of some less-aggressive tumors changes to a more aggressive nature, typically from well-differentiated papillary or follicular thyroid carcinoma to poorly differentiated carcinoma or anaplastic thyroid carcinoma [14]. However, a similar change in MTC is not well documented. Patients with MEN 2A or FMTC, on the other hand, often show the following interesting and contradictory phenomena: onsets at young ages, frequent nodal metastases, persistent hypercalcitoninemia postoperatively indicating persistent disease, but relatively good survival and long-term survival even in patients with distant metastases, as we describe below in Materials and methods section. In light of these interesting and contradictory phenomena, we hypothesized that the growth of hereditary MTCs might slow down with aging and that a minority of MTCs acquire an aggressive change over a long term.

To test our hypothesis, we calculated the Ct-DTs in the earlier and later half-periods of the surveillance in MTC patients with postoperative hypercalcitoninemia.

## Materials and methods

### Case presentation

#### Patient A: an 18-year-old woman with MEN 2A

**Fig. 1:**
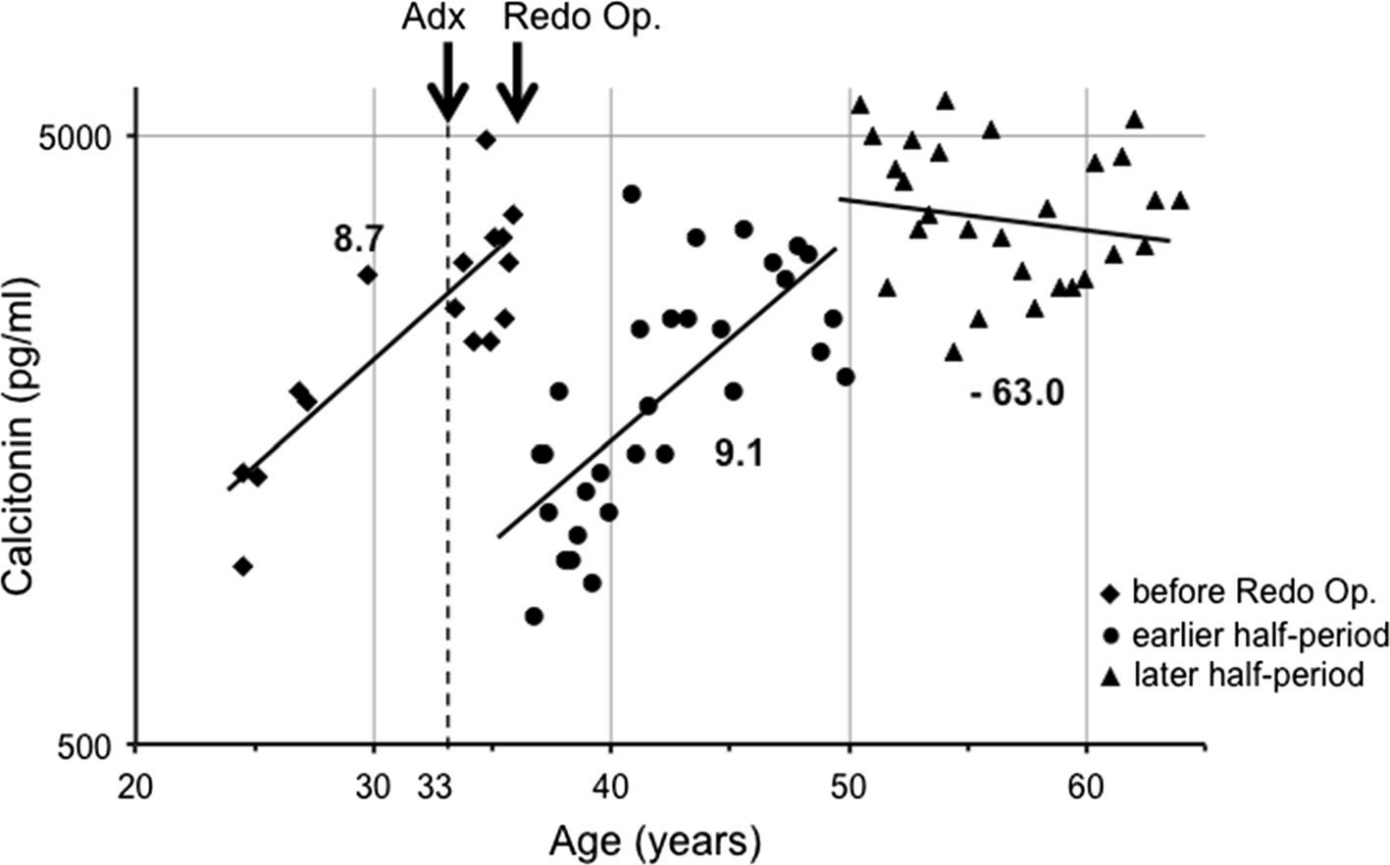
Changes in serum calcitonin levels and major clinical events in Patient A with MEN 2A. She underwent the first thyroid surgery at 18 years old for MTC, a bilateral adrenalectomy at 33 years old, and the second neck surgery for recurrent tumor at 36 years old. Note that the vertical axis for calcitonin values is shown in log scale. Lines show the regression lines in three periods: before the redo surgery, the earlier half-period, and the later half-period after redo surgery. Redo Op.: the second neck surgery for recurrent tumor. Adx: bilateral adrenalectomy for bilateral pheochromocytoma. Numbers indicate Ct-DT values (years)

In November 1969, an 18-year-old woman underwent a subtotal thyroidectomy with a right modified neck dissection for bilateral thyroid cancers. The pathological diagnosis was bilateral medullary thyroid carcinomas with multiple nodal metastases. In 1975, when the measurement of calcitonin became available, she was found to have hypercalcitoninemia indicating persistent disease (Fig. 1). However, imaging studies failed to reveal metastatic lesions. In 1984, she underwent a bilateral total adrenalectomy for bilateral pheochromocytoma through an abdominal approach. Multiple small nodules were found on her liver surface, a biopsy of which showed metastatic medullary carcinoma. In 1987, she underwent a completion thyroidectomy and left modified neck dissection and extirpation of an enlarged parathyroid gland for recurrent MTC and the appearance of primary hyperparathyroidism. In 2014, at 63 years old, she was asymptomatic with elevated serum calcitonin (3900 pg/ml) and CEA (177 ng/ml) levels. Imaging studies revealed multiple small low-density lesions with spotty calcifications in her liver, consistent with multiple small liver metastases. She carries *RET* codon 634 mutation.

The changes in her serum calcitonin levels over time and major clinical events are shown in Fig. 1. Periodic measurements of serum calcitonin following the initial thyroid surgery showed a moderate increase in the values with the Ct-DT of 8.7 years, a decrease after the second neck surgery followed by a similar increase with Ct-DT of 9.1 years, and interestingly, a significant gradual decrease in serum calcitonin levels, giving a negative value to Ct-DT at −63.0 years since approx. 45–50 years of age, without any causative events.

## Methods

From April 1969 to April 2005, 175 patients with MTC underwent surgical treatment at Kuma Hospital. Among them, patients who satisfied the following inclusion criteria were selected for the present study: patients who had obvious persistent hypercalcitoninemia postoperatively, were followed for >10 years following the initial surgery or the redo surgery if this was performed, and did not receive major interventions such as surgery or external beam radiation during the study period. Tyrosine kinase inhibitors for medullary thyroid carcinoma were not available in the present study period in Japan and were not used for the present patients.

There were 26 patients (21 females and five males) (Table 1). Fourteen had hereditary MTC (nine MEN 2A and five FMTC). Twelve patients had sporadic MTC. All 14 of the patients with hereditary MTC carried germ line *RET* mutations at codons 634, 620, 618, 611, and 768 in 8, 2, 1, 1, and 2 patients, respectively, and none of the 12 patients with sporadic MTC was found to carry the mutations (Table 1). Two of the hereditary MTC patients underwent their initial thyroid surgery elsewhere. The extent of disease progression and surgery performed for these patients are shown in Table 2. Majority but not all of the present patients had rather advanced disease such as stage IVA. Therefore, they underwent rather extensive surgery. Seven of the hereditary MTC patients and two of the sporadic MTC patients had redo surgery for recurrent disease before the present study period. None of the patients had structural disease detected on imaging studies at the beginning of the present study period except for Patient A described above.

**Table 1:** Clinical features of the 26 patients with medullary thyroid carcinoma (MTC)

	Hereditary MTC	Sporadic MTC	Significance
No. of patients, female: male	14 (12:2)	12 (9:3)	*P *= 0.490
Primary op.: Redo op.	12:2	12:0	*P *= 0.173
Phenotype: MEN 2A: MTC only	9:5	0:12	*P *= 0.001
Redo op. before the study period	7	2	*P *= 0.075
Age at first surgery (years)^a^	38 (18–76)	40.5 (32–66)	*P *= 0.227
Max. tumor size (mm)^a^	25 (14–50)	26.5 (13–50)	*P *= 0.543
Follow-up period (yrs)^a^	28.4 (10.9–45.1)	21.1 (10.6–28.4)	*P *= 0.123
Ct follow-up period (years)^a^	19.1 (10.8–30.2)	17.5 (10.6–28.4)	*P *= 0.718
No. of Ct measurements^a^	29.5 (10–77)	40 (17–91)	*P *= 0.268
Died of disease	1	2	*P *= 0.449
Died of other disease	1	1	*P *= 0.910

^a^Median (range). Ct, calcitonin; MEN 2A, multiple endocrine neoplasia type 2A; MTC, medullary thyroid carcinoma

**Table 2:** TNM stage and the initial surgery performed for the 26 patients with medullary thyroid carcinoma (MTC)

	Hereditary MTC	Sporadic MTC	Significance
TNM stage			*P *= 0.817
T1bN0M0 stage I	1	1	
T2N0M0 stage II	4	5	
T1bN1bM0 stage IVA	0	1	
T2N1bM0 stage IVA	5	4	
T3N1bM0 stage IVA	2	1	
Unknown	2	0	
Surgery			*P *= 0.082
Hemi Tx	0	1	
Hemi Tx + M	0	3	
Subtotal Tx + M	3	0	
Total Tx + M	4	2	
Total Tx + M2	5	4	
Total Tx + M + Med	0	2	
Unknown	2	0	

TNM stage according to UICC eighth edition. Hemi Tx: hemithyroidectomy, Subtotal Tx: subtotal thyroidectomy, Total Tx: total thyroidectomy, M: modified neck dissection, M2: bilateral modified neck dissection, and Med: mediastinal dissection. N.S.: not significant

The median surveillance periods for the hereditary MTC patients and the sporadic MTC patients were 28.4 years and 21.1 years, respectively. Two patients had redo surgery for recurrent disease after the study period, and three died of the disease.

Patients were seen typically twice a year with measurements of serum calcitonin, and imaging studies were added when indicated. Serum calcitonin was measured for each visit by a laboratory (SRL, Tokyo) using the solid two-site immunoradiometric assay (Mitsubishi Chemical, Tokyo). This was the only approved measurement method for calcitonin in Japan until April 2015, and the end of the present study period was set at that time.

The median Ct follow-up periods in the hereditary MTC and sporadic MTC patients were 19.1 years and 17.5 years, respectively (Table 1). The two groups’ median numbers of calcitonin measurements were 29.5 and 40, respectively. We divided each patient’s study period into the earlier and later halves and calculated the calcitonin doubling time (Ct-DT) for each period as described [9]. In brief, we calculated a regression line from the changes in Ct values over time for each period in individual patients. Then, Ct-DT was calculated with the slope (*b*) of the regression line as (log 2)/*b*.

### Statistical analyses

The statistical tests used to compare groups included Student’s *t* test, the Mann–Whitney *U*-test for skewed variables, and the Chi-square test for differences in count and frequency. As described below in Results section, some of the patients showed a decrease in serum calcitonin levels over time, giving negative values for Ct-DTs. This created a discontinuity problem between the positive Ct-DT values and the Ct-DTs in negative values. Thus, the statistical analyses on DTs were performed on the reciprocal of DT (i.e., 1/DT) as Barbet et al. described [10]. Ct-DT was calculated from the slope of the regression line of the changes in Ct values over time for the earlier and later half-periods in individual patients. We evaluated the difference between the slope of the regression line in the earlier half-period and that in the later half-period for each individual patient by using a standardized major axis (SMA) technique [15]. If the difference in these slopes in a patient was significant, the difference in the Earlier-Ct-DT value and the Later-Ct-DT value in the patient was regarded significant. Statistical analyses were performed using the program Stat Flex (View Flex, Tokyo). A *p* value <0.05 was considered significant.

## Results

**Fig. 2:**
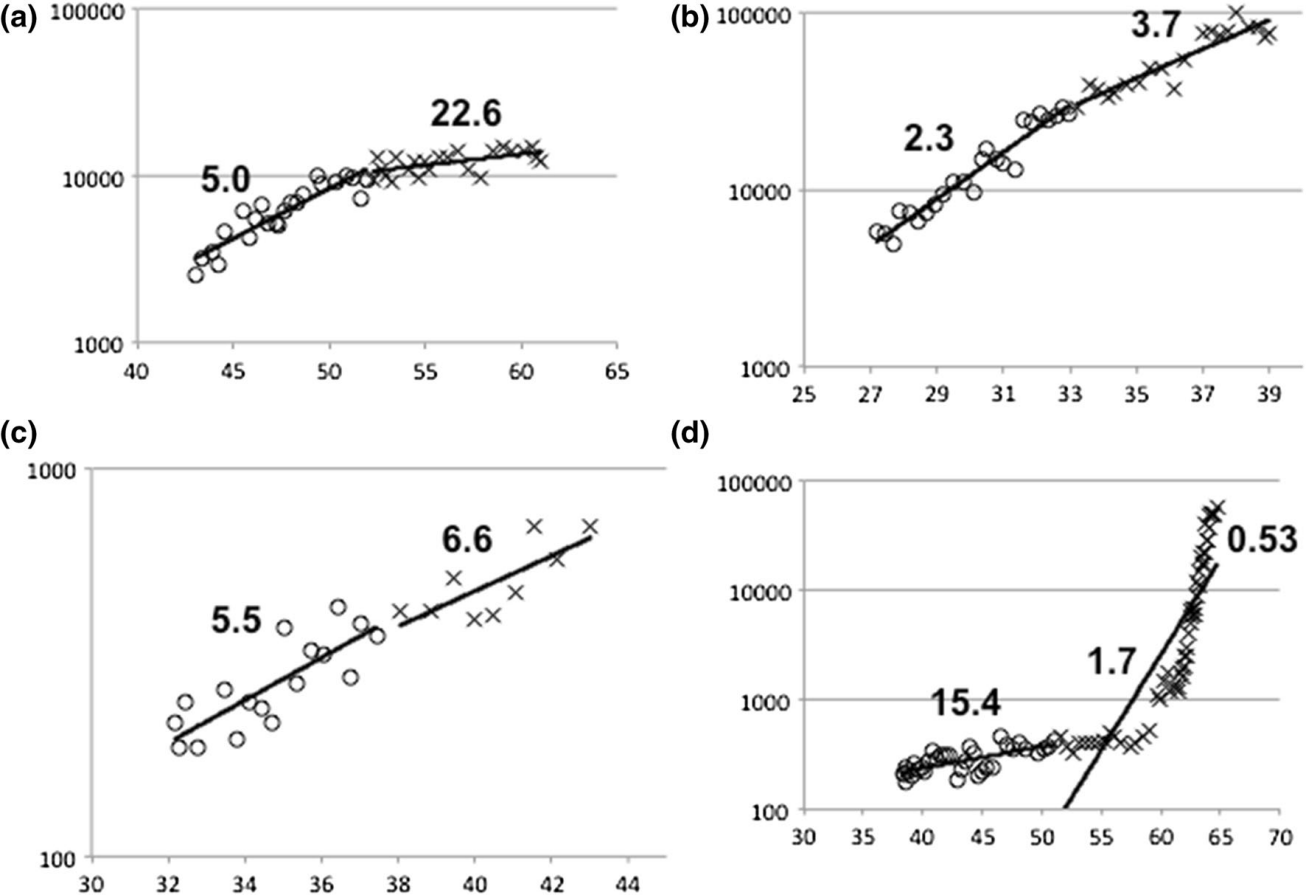
Representative cases showing changes in serum calcitonin levels over long periods. **a** A patient who had a significantly longer Later-Ct-DT (22.6 years) than Earlier-Ct-DT (4.0 years) (*p* < 0.01). **b** A patient who had a significantly longer Later-Ct-DT (3.7 years) than Earlier-Ct-DT (2.3 years) (*p* < 0.01). **c** A patient whose Later-Ct-DT (6.6 years) and Earlier-Ct-DT (5.5 years) were not significantly different (n.s.). **d** A patient who had a significantly shorter Later-Ct-DT (1.7 years) than Earlier-Ct-DT (15.4 years) (*p* < 0.01). The Ct-DT after the aggressive change was 0.53 years. The vertical axes indicate the serum calcitonin levels (pg/ml) shown on log scale. The horizontal axes indicate the patient’s age (years). Lines indicate regression lines. Numbers indicate Ct-DT (years)

Many of the present patients showed a decrease in the increase rate of their serum calcitonin level over a long period; some showed a decrease in their calcitonin levels, whereas some showed no change in the increase rate, and a few showed a sudden increase in the levels. Representative cases are shown in Figs. 1 and 2a–d.

**Fig. 3:**
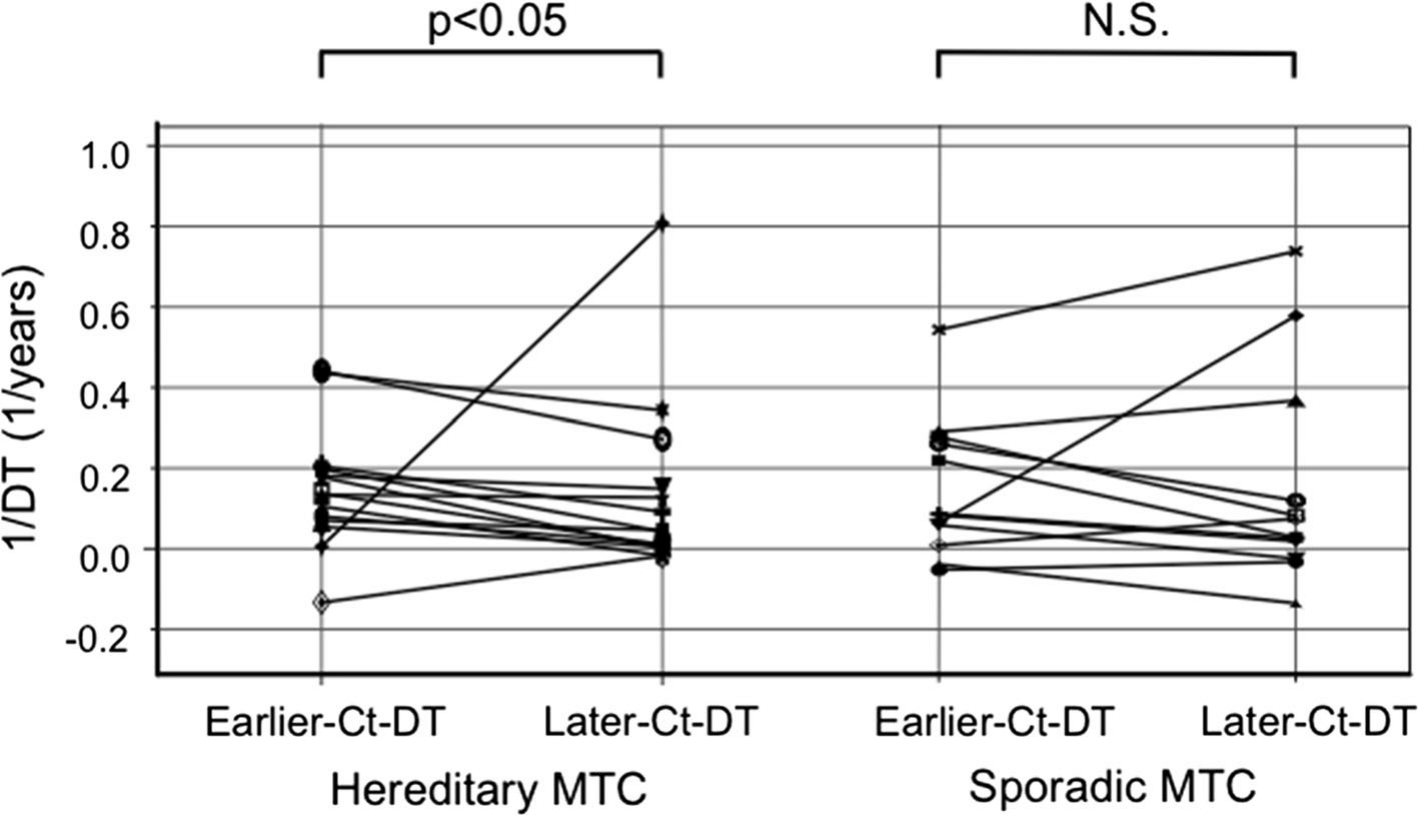
Reciprocals of the Ct-DTs in the earlier and later half-periods in the patients with hereditary MTC and sporadic MTC. The medians (range) for these values are given in Tables 2 and 3

Among the 14 hereditary MTC patients and in the earlier half-period of the study period, 13 patients showed a gradual increase in their serum calcitonin levels with the median Ct-DT of 7.2 years, and the other hereditary MTC patient showed a gradual decrease in the level with the Ct-DT of −7.6 years. In the later half-period, 12 showed a slower increase in their serum calcitonin levels with the median Ct-DT of 15.7 years, and the other two patients showed a decrease in the levels with the median Ct-DT of −62.2 years (Table 2). In this group of hereditary MTC patients, the Later-Ct-DT (median, 20.0 years) was significantly longer than the Earlier-Ct-DT (median, 7.1 years) (*p* = 0.013) (Table 3, Fig. 3), indicating that the growth rate in the later half-period was decreased compared to that in the earlier half-period. However, two of the hereditary MTC patients showed shortening in their Ct-DT indicating an increase in their tumor growth rate, as demonstrated in Fig. 3.

**Table 3:** Calcitonin doubling time (Ct-DT) in the earlier and later half-periods in the 14 patients with hereditary MTC

	Ct-DT		Significance
	Earlier period	Later period	
Surveillance period (years)	9.4 (5.2 to 14.3) (*n* = 14)	8.9 (1.1 to 14.7) (*n* = 14)	*P *= 0.748
No. of measurements	19 (4–38) (*n* = 14)	13.5 (5–39) (*n* = 14)	*P *= 0.250
Doubling time (years)			
Positive value	7.2 (2.3–117.9) (*n* = 13)	15.7 (1.2–333.0) (*n* = 12)	–
Negative value	−7.6 (−7.6) (*n* = 1)	−62.2 (−63.3 to −61.0) (*n* = 2)	–
1/doubling time (1/years)	0.14 (−0.13 to 0.44) (*n* = 14)	0.05 (−0.02 to 0.81) (*n* = 14)	*P *= 0.013
Median DT converted from the median 1/DT (years)	7.1	20.0	–

–: not applicable. Values are medium (range)

Among the 12 sporadic MTC patients and in the earlier half-period of the surveillance, ten patients showed a gradual increase in their serum calcitonin levels with the median Ct-DT of 8.0 years, and the other two patients showed a gradual decrease in these levels with the median Ct-DT of −22.9 years. In the later half-period, nine patients showed a slower increase with the median Ct-DT of 12.0 years, and the other three patients showed a decrease with the median Ct-DT of −31.5 years (Table 4). In the group of sporadic MTC patients, the Later-Ct-DT (median, 20.0 years) was longer than the Earlier-Ct-DT (median, 11.1 years); however, the difference did not reach significance (Table 4, Fig. 3). Although there was no significant difference as a group, many of the sporadic MTC patients showed elongation of Ct-DT, whereas some of them showed shortening of Ct-DT, suggesting a decrease and increase in their tumor growth rate, respectively (Fig. 3).

**Table 4:** Calcitonin doubling time (Ct-DT) in the earlier and later half-periods in patients with sporadic medullary thyroid carcinoma

	Ct-DT		Significance
	Earlier period	Later period	
Surveillance period (years)	7.5 (2.9–13.8) (*n* = 12)	7.6 (5.1–13.5) (*n* = 12)	*P *= 0.686
No. of measurements	18 (10–38) (*n* = 12)	18.5 (6–57) (*n* = 12)	*P *= 0.795
Doubling time (years):			
Positive value	8.0 (1.8–114.3) (*n* = 10)	12.0 (1.4–51.8) (*n* = 9)	–
Negative value	−22.9 (−26.5 to −19.3) (*n* = 2)	−31.5 (−41.0 to −7.4) (*n* = 3)	–
1/doubling time (1/years)	0.09 (−0.05 to 0.54) (*n* = 12)	0.05 (−0.14 to 0.74) (*n* = 12)	*P *= 0.774
Median DT converted from the median 1/DT (years)	11.1	20.0	–

Values are medium (ranges)

**Fig. 4:**
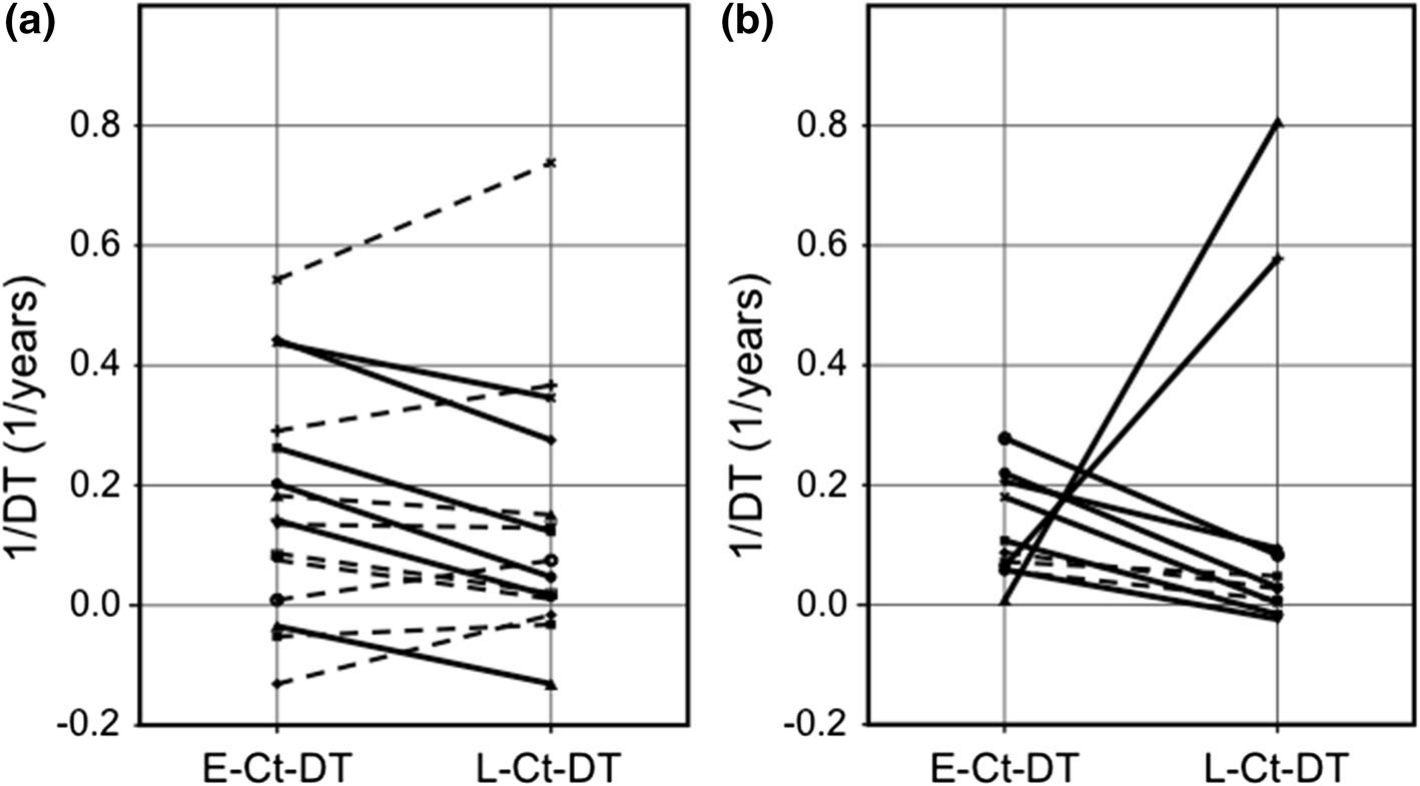
Reciprocals of Ct-DTs in the earlier and later half-periods in the patients with MTC. **a** (left panel): Patients who had 10–20 years surveillance. **b** (right panel): Patients who had >20 years surveillance. Fourteen patients who had significant differences between their Ct-DTs in the earlier and later half-periods are shown with thick lines, and 12 patients without significant differences are shown with broken lines. Eight of the 11 patients with >20 years surveillance had significant differences in these two values, while six of the 15 patients with 10–20 years surveillance had significant differences in these two values. Twelve of these 14 patients with significant differences in their earlier and later half-period values had later half-period 1/DT values smaller than their earlier half-period 1/DT values, indicating deceleration in their tumor growth rates, while the remaining two patients had later half-period 1/DT values larger than their earlier half-period 1/DT values indicating acceleration in their tumor growth

When the patients were grouped according to the length of the surveillance, among the 15 patients who had 10–20 years surveillance their Later-Ct-DTs were not significantly different from their Earlier-Ct-DTs as a group: median (ranges) of 1/DT being 0.075 (−0.135–0.738) and 0.139 (−0.131–0.543), respectively (*p* = 0.302) (Fig. 4a). While the analyses on the difference between the slope of the regression line of the changes in Ct values over time in the earlier half-period and that in the later half-period in individual patients showed that six of them had significantly longer Later-Ct-DT than his or her Earlier-Ct-DT, indicating deceleration and regression of tumor growth in these patients. Among the 11 patients with >20 years surveillance, their Later-Ct-DTs were not significantly different from their Earlier-Ct-DTs (*p* = 0.065) as a group, probably because of the presence of two patients with markedly shortened Later-Ct-DT. The remaining patients showed elongation in Later-Ct-DT (Fig. 4b).

Our analysis on the difference in the slopes of the regression lines of the changes in Ct values over time in these two periods in individual patients revealed that 12 patients (seven hereditary MTC patients and five sporadic MTC patients) had significantly longer Later-Ct-DTs compared to their Earlier-Ct-DTs (median Ct-DT: 27.4 vs. 4.9 years). Of these 12 patients, five hereditary MTC patients and five sporadic MTC patients had a Later-Ct-DT that was more than two times the value of their Earlier-Ct-DT, indicating a significant slowdown in the growth rate. On the other hand, two patients (one hereditary MTC patient and one sporadic MTC patients) had a significantly and markedly shorter Later-Ct-DT than Earlier-Ct-DT (1.2 vs. 125 years and 1.7 vs. 15.4 years, respectively) (Figs. 2d, 4b). These data indicate that 12 of the present patients had a decrease in their tumor growth rate over a long period; that is, ‘spontaneous deceleration of tumor growth.’ Three of these patients had negative Later-Ct-DT values indicating ‘regression of the tumor,’ whereas two patients had a marked shortening in their Ct-DT that might indicate ‘acceleration of tumor growth’ reflecting an ‘aggressive change’ in the nature of their tumors. These two patients developed structural recurrence in bones and lungs and died of the disease.

## Discussion

Our present findings revealed that the majority of the 14 patients with hereditary MTC who had persistent hypercalcitoninemia postoperatively showed elongation of their Ct-DTs over a long period, indicating deceleration in their tumor growth rate. Two patients showed a decrease in their serum calcitonin levels, indicating possible tumor regression. Change in serum tumor marker levels over time in a particular patient is generally regarded as a surrogate for the change in tumor mass of the patient, and the change is best expressed as serum tumor marker-doubling time. Elongation of serum tumor marker-doubling time may indicate slowing of tumor growth rate, or alternatively it may be due to decreasing production of the tumor marker by the tumor that has a constant growth. Without tumor mass data, one cannot distinguish between these two possibilities. We could not evaluate the tumor volume-doubling times, since most of the present MTC patients did not have measurable structural diseases. However, the patients with elongation of their Ct-DTs had good prognoses, and none of them died of the disease during the long-term surveillance. In addition, patients who had shortening of their Ct-DT developed structural recurrence and died of MTC.

The changes between the Earlier-Ct-DT and the Later-Ct-DT in the present 12 sporadic MTC patients did not reach significance. However, this was due to the presence of some patients who had a shorter Later-Ct-DT than Earlier-Ct-DT. Five of the 12 sporadic MTC patients did have significantly longer Later-Ct-DTs than Earlier-Ct-DTs, indicating that spontaneous deceleration of tumor growth occurred in sporadic MTCs too.

On the other hand, one patient with hereditary MTC and another patient with sporadic MTC showed shortening of their Ct-DT and both of them developed structural recurrence and died of the disease, consistent with aggressive change in their tumor nature.

Although the precise underlying mechanisms have not been established, the phenomena of spontaneous regression have been well documented in many malignant tumors such as neuroblastoma, hypernephroma, malignant melanoma, choriocarcinoma, and malignant lymphoma [16, 17]. Thus, spontaneous tumor regression and a growth deceleration might not be rare phenomena.

Mazzaferri et al. [18] reported that papillary thyroid carcinomas (PTCs) showed a unique biological behavior related to the patient’s age at surgery. Young patients showed high recurrence rates but excellent survival, and elderly patients showed high recurrence rates and high mortality rates compared to middle-aged patients [18]. These confusing age-related phenomena are not yet understood. In our study of 426 PTC patients who underwent a total thyroidectomy, the proportion of patients with detectable serum thyroglobulin indicating biochemically persistent disease was high in the patients aged <40 years and those ≥60 years and low in the patients 40–60 years. The proportion of patients with a short thyroglobulin-DT and a high Ki-67 labeling index indicating rapid tumor growth increased with age, and the proportion of PTC patients with a long thyroglobulin-DT and a low Ki-67 labeling index indicating slow tumor growth decreased with age [19, 20]. Thus, the growth of PTCs is strongly related to age. However, these were cross-sectional observations.

Here, we report, for the first time, a spontaneous deceleration of tumor growth in hereditary MTCs based on long-term longitudinal observations of the same patients. Some of the sporadic MTCs also showed a similar phenomenon. A similar spontaneous growth deceleration might occur in the PTCs of young patients; this may be demonstrated with careful longitudinal observations. If this is the case, the confusing phenomena in which PTCs in children often advance and show high recurrence rates and yet the patients show excellent survival can be understood, although the underlying mechanisms remain to be clarified.

Gain of function germ line and somatic mutations in *RET* proto-oncogene were reported as pathogenetic mutations of hereditary MTC and sporadic MTC, respectively [3, 4]. RET/PTC rearrangements and BRAF mutations were also reported in PTC [21–23]. All of these mutations are involved in the MAPK signaling pathway and activate the pathway. Age might be an important factor affecting this pathway through as-yet unknown mechanisms.

Two of the present 26 MTC patients (one hereditary and one sporadic) showed a marked shortening in their Ct-DTs over time indicating an aggressive change in their tumors’ nature, and they developed structural recurrence and died of the disease. Some MTCs may have aggressive changes over a long-term period similar to the poor differentiation or anaplastic change in differentiated thyroid cancer.

Our present analyses revealed spontaneous deceleration and possible regression in tumor growth in hereditary MTCs as well as in some of the sporadic MTCs, and aggressive change in a few of the MTCs. However, this patient series was carefully selected, based on the inclusion criteria with a surveillance period >10 years without major interventions. Patients who had recurrence and were treated for it within 10 years were not included. Therefore, the proportions of MTC patients in general who show these changes in tumor growth rates remain to be clarified. Our findings must therefore be tested in future studies, and the mechanisms underlying these phenomena also remain to be elucidated.

## Conclusion

Many of the hereditary and some of the sporadic MTC patients had elongated Ct-DTs over a long period, suggesting spontaneous deceleration and regression of tumor growth. A minority of the MTC patients showed Ct-DT shortening, suggesting an aggressive change in their tumor nature.
